# Density-matrix evaluation of the enhancement to resonant Raman scattering and fluorescence of molecules confined in metallic nanoparticle dimers

**DOI:** 10.1038/s41598-018-20328-x

**Published:** 2018-01-30

**Authors:** Yong Wei, Li Li, De-Xian Sun, Ming-Li Wang, Yan-Ying Zhu

**Affiliations:** Hebei Key Laboratory of Microstructural Material Physics, School of Science, Yanshan University, Qinhuangdao, 066004 China

## Abstract

In the present work we study the surface-enhanced resonant Raman scattering (SERRS) and fluorescence (SEF) spectra of a general model molecule confined in metallic dimers consisting of Ag, Au and hybrid AuAg nanoparticles (NPs). The electromagnetic (EM) enhancement factors were simulated by the generalized Mie scatting method and the scattering cross section of the molecules were obtained by density-matrix calculations. The influence of the size of the NPs and the separation between the dimer on the Raman scattering and fluorescence were systematically studied and analyzed in detail. It was found that the SERRS mainly related to EM enhancement and the SEF depended on the competition between EM enhancement and quantum yield, both of which could be controlled by tuning the radius and separation of the metallic dimers. The optimal radius of the NPs for SERRS were found to be around 30 nm for AgNPs, 40 nm for AuNPs and 50 nm for hybrid AuAgNPs. The strongest Raman enhancement as predicted by the theoretical simulations were 6.2 × 10^10^, 1.5 × 10^7^ and 5.2 × 10^8^ for the three types of structures, respectively. These results could offer valuable information for the design of metallic substrates for surface enhanced Raman and fluorescence measurements.

## Introduction

Recently, with the development of single-molecule detective technology, surface-enhanced resonant Raman scattering (SERRS) has become a valuable tool for surface science, medical diagnostics and biomedical applications due to the ultrahigh sensitivity and stability^1–5^. The localized surface plasmon resonance (LSPR) can enhance the interaction between incident light and metallic nanostructures, and thus, the enhancement in the vicinity of metallic nanostructures mainly results from strong electromagnetic (EM) coupling effect^6–11^. It is commonly accepted that the SERRS process composes two parts: the EM enhancement to the incident laser and the emission enhancement of the scattered photons^12^. As a result, the Raman cross section of a molecule near metallic nanoparticles (NPs) will be enhanced by the fourth power of the EM enhanced factor *M*^13,14^. With such great enhancement to the signal, SERRS can be used to detect samples at extremely low concentrations, even reach the single-molecular level^15,16^. At the same time, the LSPR can also affect the fluorescence intensity, a process normally termed as surface enhanced fluorescence (SEF). Unlike the SERRS intensity, the SEF intensity for high quantum yield molecules normally varies as the second power of the electromagnetic enhancement. In addition, the SEF enhancement is relative weak due to the consequence of competition between the enhancing and quenching when the molecule is close to the NPs. In general, both SERRS and SEF are crucially dependent on the nano-substrate. The most commonly used substrates are noble metal NPs, such as Ag and Au, because of their excellent LSPR activity and relatively simple preparation process^17–19^. For the metallic dimer configuration, the EM enhancement (*M*) and scattering cross section are related to many factors, such as the material of the substrate, the size of the NPs, the separation between NPs, as well as the laser photon energy. All of these factors have to be considered in the actual design of active substrate which could become quite complicated in the experiment. To this end, a detailed theoretical analysis on such systems could provide valuable information in obtaining the optimal substrates.

In this paper, we use the density-matrix approach developed by Xu *et al*. to calculate the surface-enhanced Raman scattering and fluorescence of a Rhodamine 6 G (R6G) molecule confined in metallic dimers. The EM enhancement to the incident laser field and the enhancement of the metallic dimer on the radiative and non-radiative decay rates of the molecule were both computed with the generalized Mie theory^20^. The non-local effect, which was mainly caused by creation of electron-hole pairs, was also account for with the d-parameter methods described by Johansson *et al*.^21^. The d-parameters for the metals were taken from ref.^22^. With the density-matrix approach, the surface enhanced Raman and fluorescence spectra of Rhodamine 6G (R6G) molecule using AgNPs, AuNPs and hybrid AuAgNPs as substrate were simulated and analyzed. Moreover, the influence of EM enhancement and quantum yield on SERRS and SEF spectrum was also investigated in detail. By comparing the scattering spectra of AgNPs, AuNPs and hybrid AuAgNPs with different radius and separation, the optimal configurations of the three substrates were presented for improving the accuracy and sensitivity in actual testing.

## Results and Discussion

**Figure 1:**
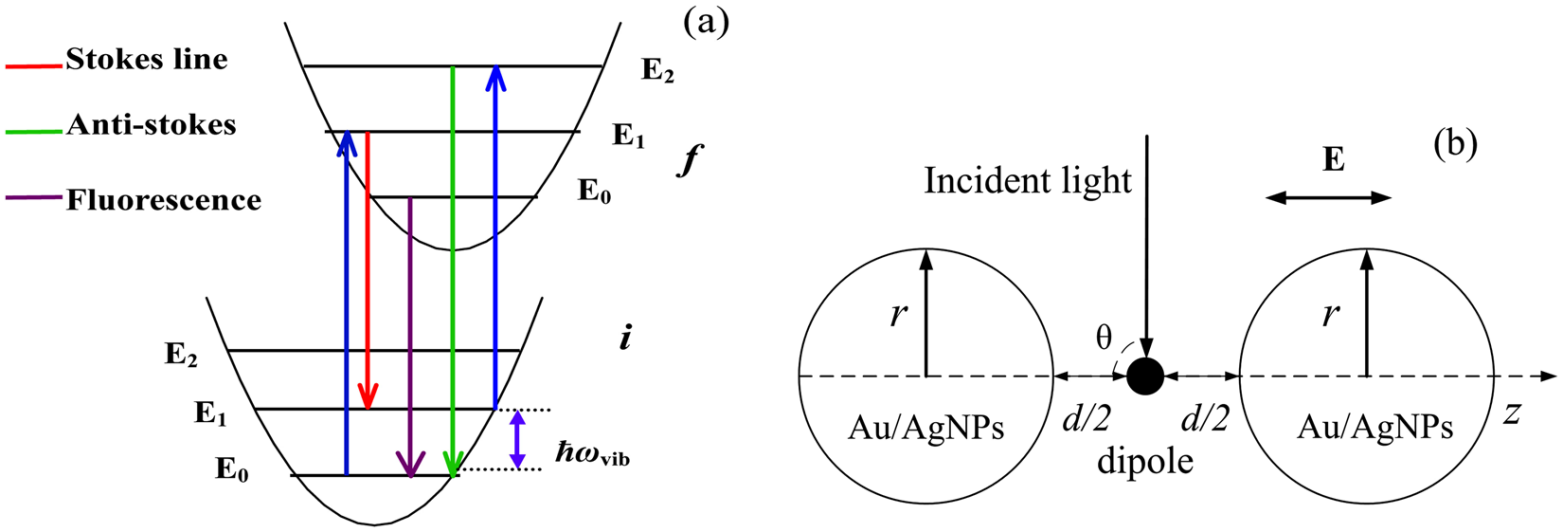
(**a**) Schematic illustration of the energetic diagrams describing the resonant Raman and fluorescence of molecule. Here we consider only the ground state and the first excited state of the molecule. (**b**) Schematic drawing of the setup for the electromagnetic simulations. The molecule is described as an oscillating dipole and is placed in the middle of the gap between the NPs.

In the simulations, we describe the electronic degrees of freedom of the R6G molecule with a two-state model, as shown in Fig. 1a, with transition dipole moment *p*. The potential energy surfaces (PESs) of the two states are assumed to be harmonic and have the same curvature, *i.e*., no frequency difference between these two states. The molecule is deposited between two metallic NPs with separation of 2*d*^21^. The system is illuminated by a laser with polarization direction *z* and angular frequency *ω*_*in*_, as shown in Fig. 1b.

**Figure 2:**
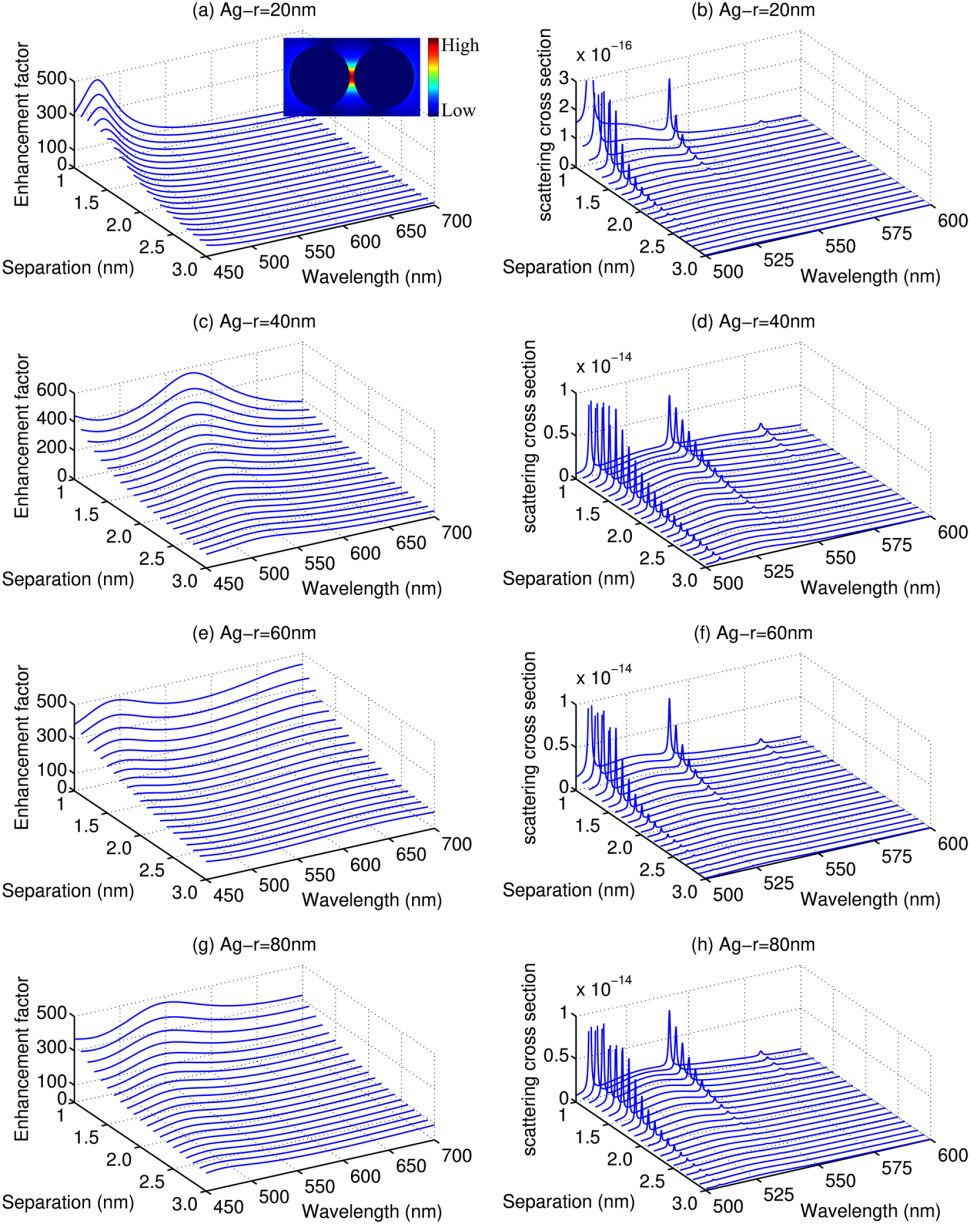
The enhancement factors (left) and scattering cross section (right) for R6G molecule placed symmetrically between two AgNPs with different radius and separations. The energy of the incident laser is 2.45 eV. (**a**,**c**,**e** and **g**) Are the enhancement factors with *r* = 20 nm, 40 nm, 60 nm, and 80 nm, respectively, (**b**,**d**,**f**,**h**) are the scattering cross section with *r* = 20 nm, 40 nm, 60 nm and 80 nm, respectively. The insert of (a) shows the typical electric field distribution of the AgNPs dimer.

Firstly, the EM enhancement factors *M* and scattering cross sections of R6G molecule placed in the middle of AgNPs dimers are simulated and compared in Fig. 2. In order to study the influence of the geometric factors of the NP dimers, the radius of AgNPs (*r*) have been varied from 10 nm to 200 nm with interval 10 nm, and the separation (*d*) are changed from 1.0 nm to 3.0 nm with interval 0.1 nm. Figure 2 shows eight sets of representative enhancement factors *M* and scattering cross section for AgNPs with r = 20 nm, 40 nm, 60 nm, 80 nm, respectively. The typical electric field distribution of the AgNPs dimer is shown in the insert of Fig. 2a. We find that the electromagnetic enhancement factor *M* is decreased and broadened rapidly with the increase of the distances between the two NPs, as shown in Fig. 2a,c,e and g. Meanwhile, we can also find that a decrease of *d* causes a clear red-shift to the resonance peaks caused by the LSPR. *M* is apparently affected by the radius *r*, and there is a larger fluctuation with *r* = 20 nm and *r* = 40 nm. Moreover, *M* is relative large in the range of wavelength longer than 500 nm with *r* = 40 nm, 60 nm, 80 nm, which is very advantageous for SERRS effect for the R6G molecule.

In the simulation of the scattering cross sections, we have set the energy of the incident laser to 2.45 eV (*λ* = 506 nm), hence, the peak at 506 nm is attributed to Rayleigh scattering, and the characteristic Raman peaks at 541 nm and 582 nm are attributed to the fundamental (0–1) and overtone (0–2) transitions, as shown in Fig. 2b,d,f,h, notice that here part of the Rayleigh peak is cut off in order to highlight the Raman peaks. It can be seen that Raman peaks are relatively high and clear with smaller *d*, especially for *r* in the range of 40 nm and 80 nm, which demonstrates that AgNPs with radius in this range are quite active for SERRS. The scattering cross section varies when *r* or *d* is changed. By increasing *d* from 1.0 nm to 3.0 nm, the scattering cross section decreases gradually, which is mainly due to the weakening of the enhancement factors *M*. Here, we are mainly interested in the resonant Raman process where the frequency of the incident laser was tuned to match the energy of a particular (real) excited state. Under such conditions, SERRS mainly consists of an excitation and an emission process: firstly, the molecule absorbs the energy of the incident photon and goes into the resonant excited state. Then, a scattered photon is emitted while the molecule returns to a different vibrational state of the ground electronic state^9^. It should be mentioned that the Raman scattering process is different with the typical fluorescence process because the former does not involve the (non-radiative) vibrational decay from higher vibrational energy levels to the vibration energy levels with lower energy in the excited state. As discussed by Johansson *et al*.^21^, both of these processes will be enhanced with enhancement factors caused by the coupling of LSPR with incident laser (${|M({\omega }_{in})|}^{2}$) and Raman scattering light (${|M({\omega }_{R})|}^{2}$), respectively. For example, the Raman peaks at 541 nm with smaller *d* are obviously stronger than others due to relatively larger *M*(*ω*_*in*_) and *M*(*ω*_*R*_).

**Figure 3:**
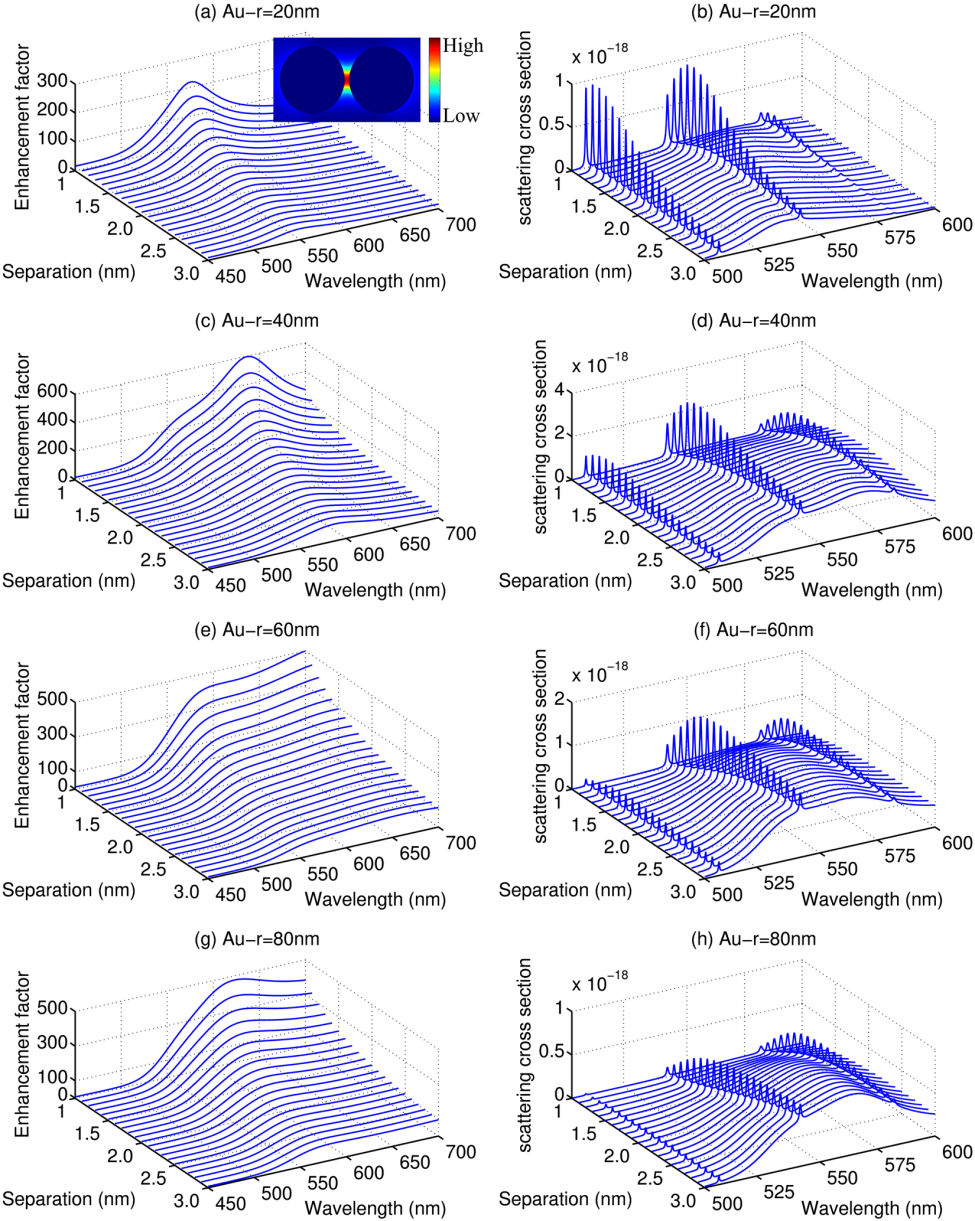
The enhancement factors (left) and scattering cross section (right) for R6G molecule placed symmetrically between two AuNPs with different radius and separations. The energy of the incident laser is 2.45 eV. (**a**,**c**,**e** and **g**) Are the enhancement factors with *r* = 20 nm, 40 nm, 60 nm, and 80 nm, respectively, (**b**,**d**,**f** and **h**) are the scattering cross section with *r* = 20 nm, 40 nm, 60 nm and 80 nm, respectively. The insert of (**a**) shows the typical electric field distribution of the AuNPs dimer.

Figure 3 shows the calculated enhancement factors *M* and scattering cross sections of the molecule confined in AuNPs dimers with different *r* and *d*. As shown in Fig. 3a,c,e,g, the enhancement factors are also sensitive to the separation *d*. When the two AuNPs approach each other, *M* grows rapidly and the resonance peaks have a clear red-shift similar to that has been observed for AgNPs. The enhancement factor *M* of the AuNPs changes more dramatically than that of the AgNPs with the increase of the wavelength, especially for *r* = 40 nm, 60 nm and 80 nm. Another noticeable difference between Au and Ag NPs is that the positions of the resonance peaks for AuNPs are red-shifted, which, on one hand is advantageous for the scattering enhancement (${|M({\omega }_{R})|}^{2}$) the of R6G molecule but has much smaller excitation enhancement (${|M({\omega }_{in})|}^{2}$). As a result, a 2–4 orders of magnitude decrease of the scattering cross section than those obtained with AgNPs is predicted for the AuNPs dimers as shown in Fig. 3b,d,f,h. Also caused by the red-shifted resonant peaks of the LSPR, the intensities of the two Raman peaks at 541 nm and 582 nm are more identifiable. Especially, for radius in the range of 40 nm–80 nm, the intensities of the Raman peaks are even stronger than the Rayleigh peak. Overall, the enhancement factor *M* is also affected by the radius *r*, as has been seen for the AgNPs, and the maximum value is obtained for AuNPs around 40 nm. Comparison of the Raman peaks indicates that, as expected, the maximum peaks are also obtained at radius of 40 nm due to the relative higher *M*.

**Figure 4:**
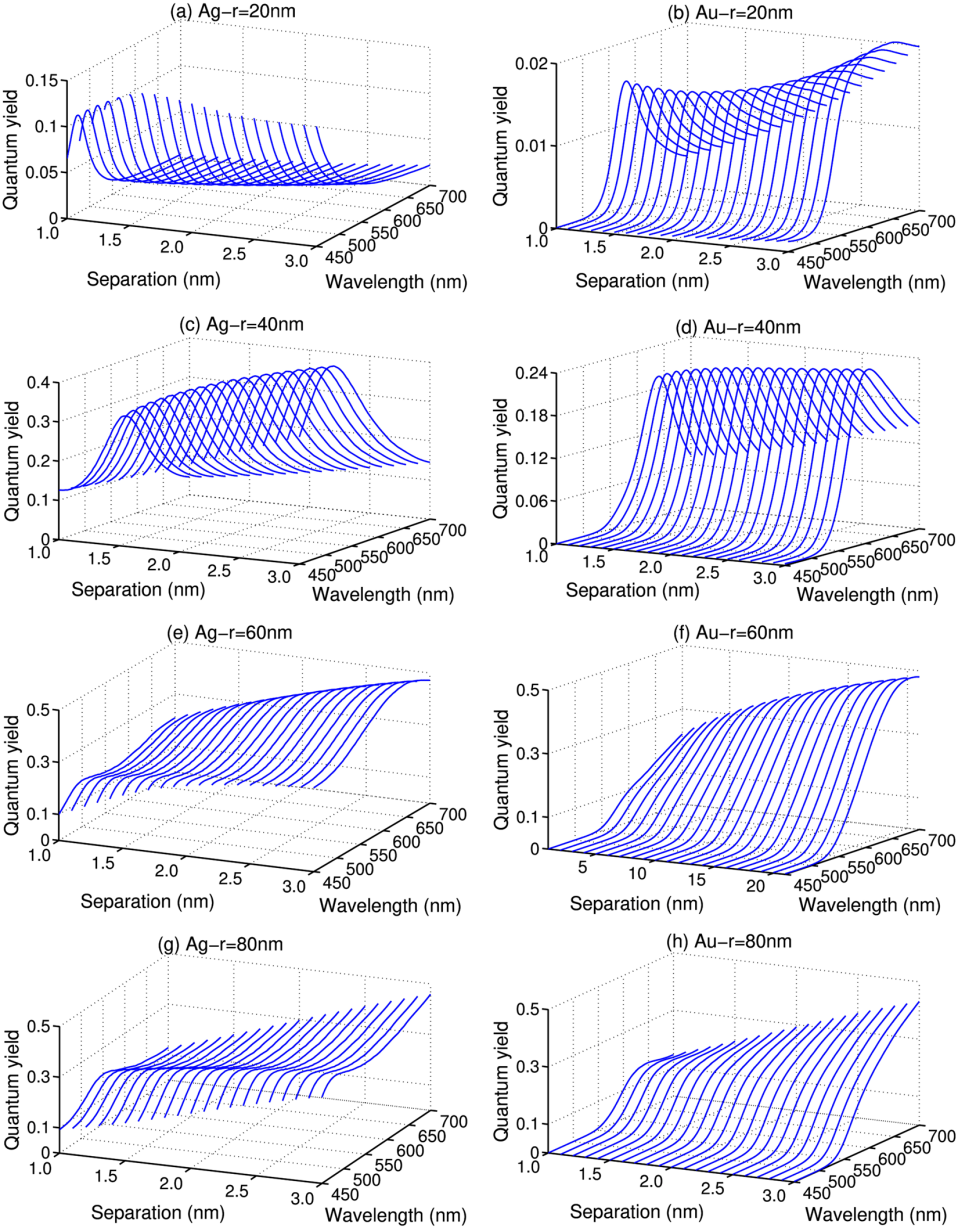
The quantum yield as a function of separation *d* with *r* = 20 nm, 40 nm, 60 nm, and 80 nm, respectively. (**a**,**c**,**e** and **g**) AgNPs, (**b**,**d**,**f** and **h**) AuNPs.

For the system studied in the present work, the resonant Raman and fluorescence spectra are always overlapped, and thus the SERRS will be affected by the SEF process. It is widely accepted that the SEF effect mainly arises from the two processes: the enhancement of excitation magnitude by local enhancement factor *M* and the enhancement of emission magnitude due to the balance of radiative and non-radiative decay rates (correlated to the quantum yield)^23–26^. Both of these factors should be influenced by the configuration of metal NPs. Figures 2 and 3 show that there is a considerably broad fluorescence background in the calculated cross sections. Meanwhile, it is noticed that the fluorescence intensities obtained with AgNPs decrease gradually with the increase of *d*, as shown in Fig. 2. However, as shown in Fig. 3, the increase of *d* actually leads to a clear increase, in certain cases followed by a slightly decrease, to the fluorescence intensities obtained with the AuNPs. Such different trends illustrate that the SEF is strongly dependent on *d* as well as the constitution of the NPs. In order to explain the SEF effect in detail, we have analyzed the quantum yield of the two types of NPs in Fig. 4. It can be seen that the quantum yields for the fluorescence always increase with the increase of *d* in the SEF range of the spectrum. However, as we have already seen in Figs 2 and 3, the enhancement factor *M* undergoes a constant decrease by enlarging the separation of the metal dimer. For the AgNPs, the increase of the quantum yield is not sufficient to compensate the reduced electromagnetic enhancement factors and, as a result, causes the fluorescence intensities to decrease with *d* in the range studied here. For the AuNPs, on the other hand, the decrease of the electromagnetic enhancement factors is not as dramatic as for the AgNPs, and the competition between the quantum yield and the electromagnetic enhancement factor and thus leads to enhancement to the fluorescence.

**Figure 5:**
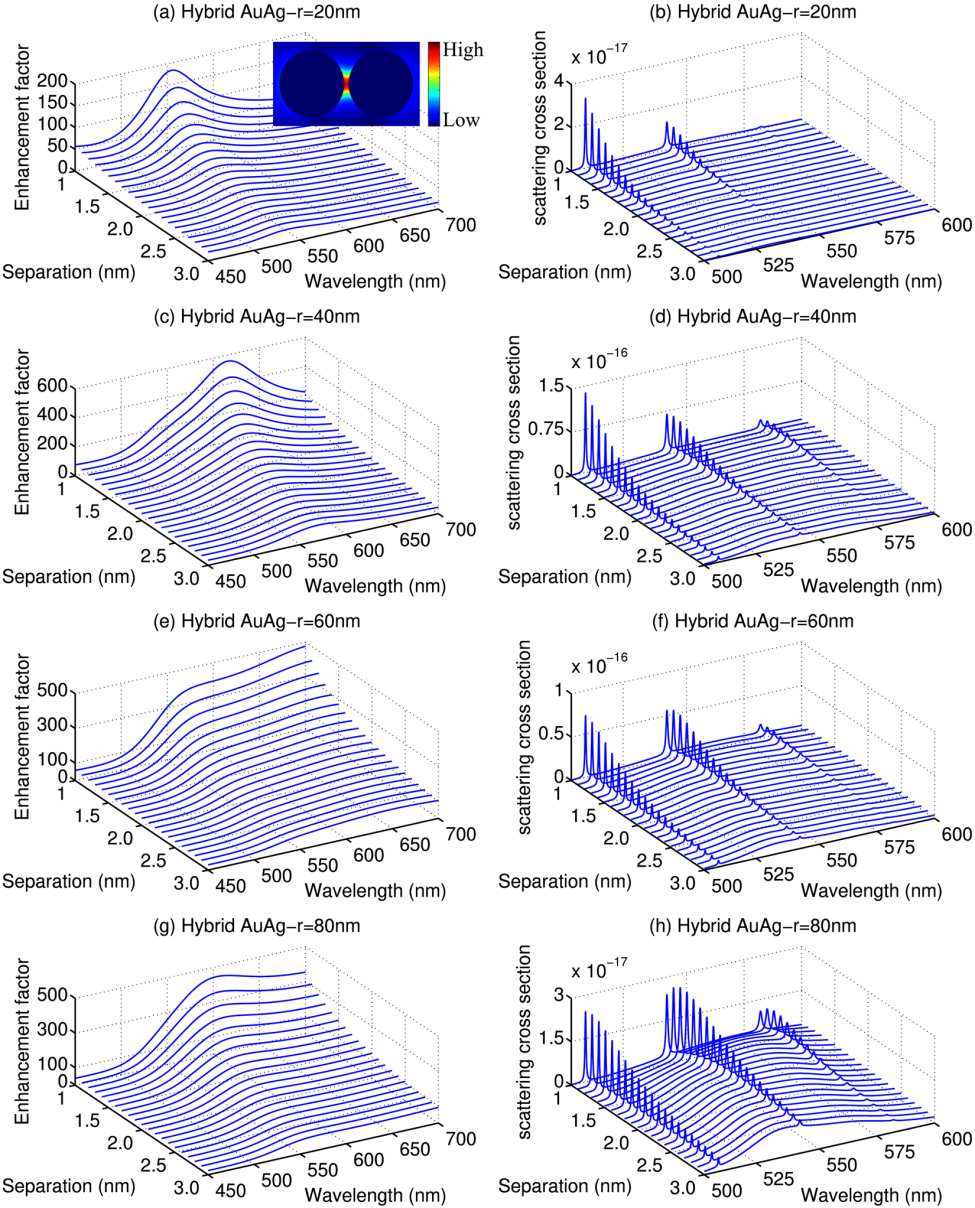
The enhancement factors (left) and scattering cross section (right) for R6G molecule placed symmetrically between two hybrid AuAgNPs with different radius and separations. The energy of the incident laser is 2.45 eV. (**a**,**c**,**e** and **g**) are the enhancement factors with *r* = 20 nm, 40 nm, 60 nm, and 80 nm, respectively, (**b**,**d**,**f**,**h**) are the scattering cross section with *r* = 20 nm, 40 nm, 60 nm and 80 nm, respectively. The insert of (**a**) shows the typical electric field distribution of the AuAgNPs dimer.

Based on the above results, we could find that the AgNPs have the better enhancement to the overall Raman and fluorescence intensities while the AuNPs offer good enhancement to the Raman overtone and the fluorescence at longer wavelength. Considering the differences of these two types of metallic dimers, we also investigated the EM enhancement and scattering cross sections of the R6G molecule confined in hybrid AuAgNPs dimers, as shown in Fig. 5. Similar to the AuNPs and AgNPs, *M* for the hybrid dimers also decreases and broadens with the increase of *d*, as can be seen in Fig. 5a,c,e,g. The energies of the characteristic LSPRs are now between the corresponding LSPRs of the pure AgNPs and AuNPs. Meanwhile, Fig. 5b,d,f,h show that the overall enhancements of the NPs to the scattering cross sections are also lies between the ones for AgNPs and AuNPs. More interestingly, very clear Raman peaks, including the overtone peaks, with relatively small fluorescence background can be seen with these hybrid NP dimers. It can also be seen that the Raman peaks are very clear with smaller separation, especially for radius from 40 nm to 80 nm due to the stronger enhancement factor. These features demonstrate that the hybrid AuAgNPs dimers are also suitable for the SERRS detection.

**Figure 6:**
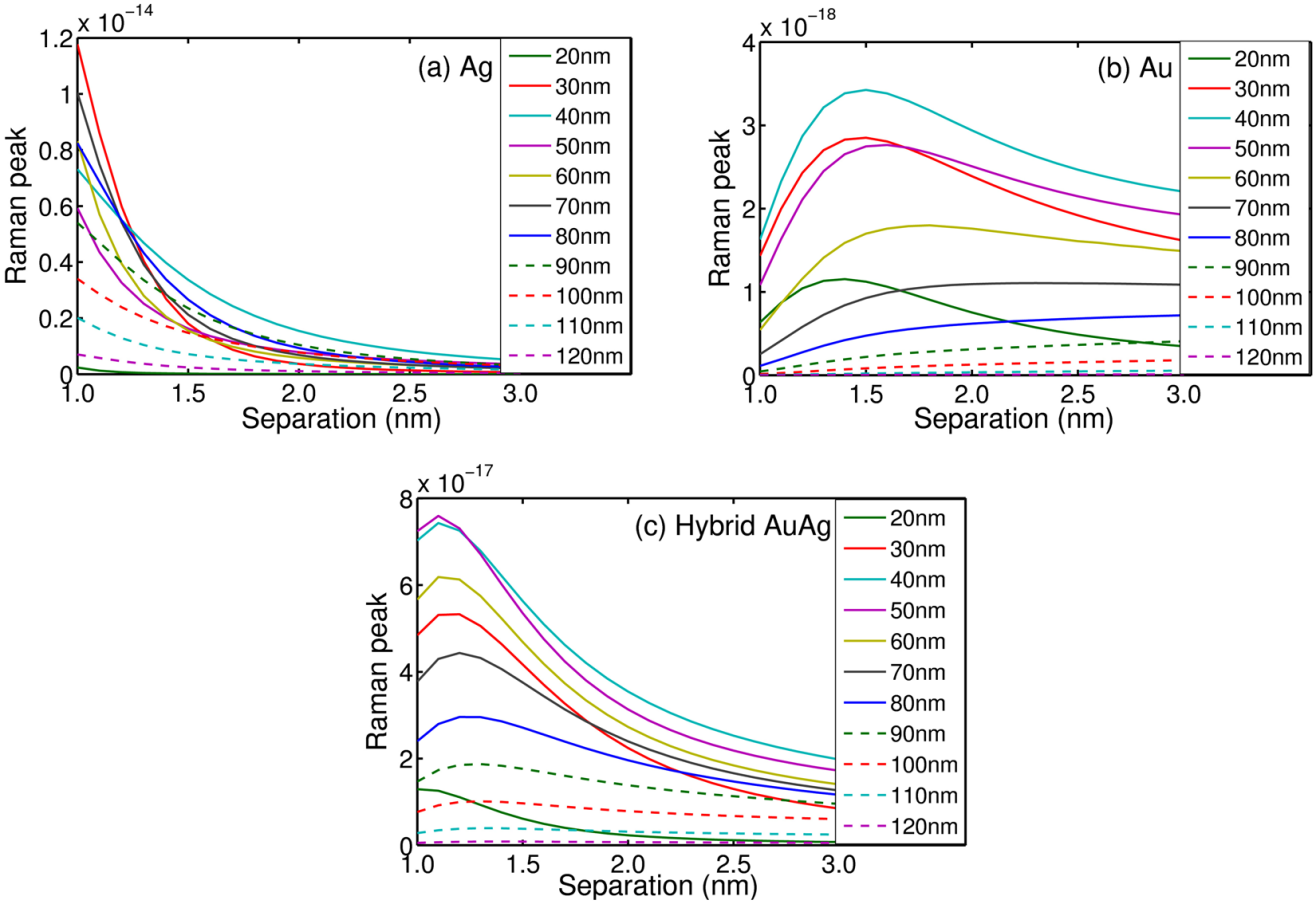
The Raman characteristic peaks of R6G molecule as a function of radius and separation at 2.29 eV (**a**) for AgNPs with radius from 20 nm to 120 nm, (**b**) for AuNPs, (**c**) for hybrid AuAgNPs

According to the above analysis, there are obvious differences among the shape and intensity of the scattering spectra obtained with AgNPs, AuNPs and hybrid AuAgNPs. To study and compare the scattering spectra in detail, here we take the first Raman peak at 2.29 eV (541 nm) as reference to study the dependence of the Raman peak on the radius and separation of the NPs. The results are given in Fig. 6. Notice that, here we consider only the radius between 20 nm and 120 nm because the Raman signals are too weak for other radius. For AgNPs, as shown in Fig. 6a, the increase of the separation causes an approximately exponential decay to the intensity of the Raman peak. And it is the smallest separations that lead to the largest Raman enhancement. It should be noted, however, the quantum effect has to be taken into account with smaller gap distances which could affect the Raman enhancement. For the systems studied in this work, we could see that AgNPs with radius of 30 nm offers the best Raman enhancement at smaller separations (*d* < ~1.3 nm), and slightly larger AgNPs (*r* = 40 nm) is the better SERRS substrate at larger separations (*d* > ~1.3 nm). The largest Raman enhancement is predicted to reach 6.2 × 10 for AgNPs with *r* = 30 nm and *d* = 1 nm. The Raman enhancement for AuNPs is 3–4 orders of magnitude smaller than that of the AgNPs with the best enhancement to be ~1.5 × 10^7^ (*r* = 40 nm, *d* = 1.1 nm), as shown in Fig. 6b. However, the dependence of the Raman cross sections on the separation is no longer the monotonic decrease as we have seen for AgNPs. Instead, a clear increase of the Raman cross section with the increase of *d* can be seen from Fig. 6b. For smaller AuNPs, the increase is followed by a decrease and a clear maximum can be found for AuNPs with *r* < 70 nm. For large NPs (*r* > 70 nm), on the other hand, the Raman cross sections are constantly increasing with the increase of *d*, a phenomenon opposite to what have been shown in Fig. 6a for AgNPs. This is mainly due to the results of the relative stronger fluorescence background at the larger separations for AuNPs. For hybrid AuAgNPs, as shown in Fig. 6c, the dependence of the Raman cross sections on the separation lies in between the ones for the pure AgNPs and AuNPs because of the mixed nature of the dimer. The maximum Raman peak is obtained when *r* = 50 nm with *d* = 1.1 nm, and the corresponding Raman enhancement is 5.2 × 10^8^, which is also in between of the optimal values for AgNPs and AuNPs. Besides, the data presented in Fig. 6 indicates that the peaks are relatively stronger when the radiuses are from 30 nm to 80 nm for the three dimers.

The configuration of the plasmonic NPs is crucial for SERRS performance, with the development of plasmonic colloidal synthesis, controllable radius and separation of spherical AgNPs and AuNPs could be obtained by a variety of novel chemical methods^27,28^. For example, the controllable radius and separation of AuNPs are synthesized by adjusting the addition amount of sodium citrate and bifunctional linker molecules or stimuli-responsive polymers^29–31^. The above results thus supply valuable information for the optimal geometrical properties of different metallic dimers for SERRS which could be helpful for the experimental synthesize of SERRS substrates.

## Conclusions

In this paper, the EM enhancement factor and SERRS cross sections of R6G molecule confined in AgNPs, AuNPs and hybrid AuAgNPs dimers are simulated and compared systematically by taking into account different particle radius and separations. Our study explains theoretically how the SERRS spectra develops as EM enhancement is varied, meanwhile, we also analyze the impact of the quantum yield and EM enhancement on the SEF. Moreover, the radius and separation dependence of the Raman peak are analyzed in detail. It is found that AgNPs have the better enhancement to the overall Raman and fluorescence intensities while the AuNPs offer good enhancement to the Raman overtone and the fluorescence at longer wavelength, and hybrid AuAgNPs, lying between the ones for AgNPs and AuNPs, have very clear Raman peaks with relatively small fluorescence background. The optimal particle radius and dimer separation for SERRS are also theoretically predicted which could be helpful for the synthesis of the metallic dimers for SERRS and SEF measurement. In addition, the radius of the relatively stronger peaks is in the range of 30 nm to 80 nm for the three dimers. Our work provides useful references for actual detection of SERRS and SEF at the nanoscale level.

## Methods

The theoretical framework to compute the SEF and SERRS has been derived by P. Johansson *et al*. in ref.^21^, here we only give a brief description of the procedure. For the case of a two-level system with ground state |*i*〉 and excited state |*f*〉 shown in Fig. 1a, the molecular dipole operator *p(r)* can be written as^19^

1 $$\begin{array}{c}{\bf{p}}({\bf{r}})=\sum _{\nu ^{\prime} }\sum _{\nu }\langle f\nu ^{\prime} |{\bf{p}}({\bf{r}})|i\nu \rangle |f\nu ^{\prime} \rangle \langle i\nu |+\sum _{\nu ^{\prime} }\sum _{\nu }\langle i\nu |{\bf{p}}({\bf{r}})|f\nu ^{\prime} \rangle |i\nu \rangle \langle f\nu ^{\prime} |\\ \qquad =\,{{\bf{p}}}^{-}({\bf{r}})+{{\bf{p}}}^{+}({\bf{r}})\end{array}$$

where **r** = (*r*, *θ*, *φ*) represents the molecular position. The operators ${{\bf{p}}}^{-}({\bf{r}})$ corresponds to the molecular absorption from the electronic ground state to the excited state. ${{\bf{p}}}^{+}({\bf{r}})$, on the other hand, represents the emission originating from the excited state to the ground state. In order to simulate the scattering and fluorescence cross section, the dipole-dipole correlation function $\langle {{\bf{p}}}^{-}({\bf{r}},t){{\bf{p}}}^{+}({\bf{r}},t+\tau )\rangle$ must be computed. It has been shown by Johansson *et al*. that the correlation function can be evaluated approximately by solving the equation of motion of the molecular density matrix^21^

2 $$i\frac{d\rho (t)}{dt}=\frac{1}{\hslash }[{H}_{0}+H\text{'},\rho (t)]+{L}_{1}\rho (t)+{L}_{2}(t)$$

where *ρ*(*t*) is the density matrix, $\rho (t)=|\psi (t)\rangle \langle \psi (t)|$. *H*_0_ is the Hamiltonian of the molecule, $H^{\prime} ={\bf{p}}\cdot {\bf{E}}({\bf{r}},t)$ describes the interaction between the laser field **E**(**r**,*t*) and the molecule. Here we have adopted the Born-Oppenheimer and Condon approximations. As a result, the electronic part of the transition dipole elements *p*_0_ is constant, and the Herzberg-Teller term and higher order terms are neglected. Within the Harmonic approximation, the Franck-Condon factor $\langle \nu ^{\prime} |\nu \rangle$ can be computed as^32–34^:

3 $$\langle \nu ^{\prime} |\nu \rangle ={(-1)}^{\nu -\nu ^{\prime} }{e}^{-\frac{S}{2}}{S}^{\frac{\nu -\nu ^{\prime} }{2}}\sqrt{\frac{\nu ^{\prime} !}{\nu !}}\sum _{k=0}^{\nu ^{\prime} }\frac{\nu !{(-S)}^{k}}{(\nu ^{\prime} -k)!(\nu -\nu ^{\prime} +k)!k!}(\nu \le \nu ^{\prime} )$$

4 $$\langle \nu ^{\prime} |\nu \rangle ={(-1)}^{\nu ^{\prime} -\nu }\langle \nu |\nu ^{\prime} \rangle (\nu  > \nu ^{\prime} )$$

where *S* is the Huang-Rhys factor.

When the molecule is placed between the NPs, the incident and scattering field will both be enhanced by the LSPPs^21^. For sphere NPs considered in the present work, the plasmonic enhancement factors (*M*) can be computed by the generalized Mie theory as^21^

5 $$M=|E(r)|/|{E}_{0}(r)|$$

with *E*(*r*) and *E*_0_(*r*) being the amplitudes of the local and incident electric field, respectively. The term ${L}_{1}\rho (t)$ in Eq. 2 describes the effective population damping. For a molecular near metallic NPs, the spontaneous emission rates are enhanced by the Purcell factor *M*_*d*_, which is given by^21^

6 $${M}_{{\rm{d}}}=\sqrt{P/{P}_{0}}$$

here *P* indicates the total radiated power when the molecular is placed near NPs, and *P*_0_ corresponds to that in the free space. The last operator ${L}_{2}\rho (t)$ in Eq. 2 is introduced to describe the dephasing effect, which is responsible for the broadening of the scattering and emission peaks.

In the simulations we solve the equation of motion of the density matrix in the rotating wave approximation. And, as shown by P. Johansson et al., the dipole-dipole correlation function can then be evaluated with the help of the quantum regression theorem^21^. The surfaced enhanced Raman scattering and fluorescence cross section can then be computed as^21^

7 $$\frac{{d}^{2}\sigma }{d\,{\rm{\Omega }}\,d(\hslash \omega )}={|M|}^{2}\frac{{\omega }^{4}{\sin }^{2}\theta }{8{I}_{in}{\pi }^{3}{c}^{3}{\varepsilon }_{0}\hslash }\mathrm{Re}{\int }_{0}^{\infty }d\tau {e}^{i\omega \tau }\langle {{\bf{p}}}^{-}({\bf{r}},t){{\bf{p}}}^{+}({\bf{r}},t+\tau )\rangle $$

where *I*_*in*_ and *ω* are incident intensity and scattering frequency, respectively. The observation angle *θ* is set to 90° in our simulation.

In the calculations, we consider a R6G molecule which is a well-studied system for Raman measurement. The molecule is assumed to be deposited between two metal NPs, and the system is illuminated by a *p*-polarized wave with polarization direction *z* and angular frequency *ω*_*in*_, as shown in Fig. 1b. The coordinates of the two NPs with radius *r* and separation *d* are *z*_1_ = (*r* + *d*/2) and *z*_2_ = (*r* + *d*/2), respectively, and the R6G is placed in the middle of the gap between the NPs. The incident photon energy and dipole moment length are $\hslash {\omega }_{in}=2.45eV$, ${p}_{0}=1.2\mathop{{\rm{A}}}\limits^{{\rm{o}}}$, respectively. The energy difference of the two states is $\hslash {\omega }_{if}=2.35eV$ and the vibration frequency is $\hslash {\omega }_{vib}=0.16eV$. In the simulations we have included four vibrational levels for each electronic states, which is sufficient to describe the most important vibrational transitions for the model R6G molecule (with Huang-Rhys factor 0.25) studied in the present work, as shown in ref.^21^. The Franck-Condon factors are computed with the DynaVib software^35^. The vibrational decay constants are chosen as ${{\rm{\Gamma }}}_{vib}^{i}=2\times {10}^{12}{s}^{-1}$ and ${{\rm{\Gamma }}}_{vib}^{f}=10\times {10}^{12}{s}^{-1}$. Meanwhile, the dephasing rate is ${{\rm{\Gamma }}}_{ph}=1.3\times {10}^{14}{s}^{-1}$ that mainly determines the width of Raman peak. Besides, in our model simulations, the separation of the NPs is greater than 1.0 nm in which the electronic potential between NPs is characterized by a large potential barrier that can effectively prevent electron tunneling. As a result, the electron tunneling effect is so weak that was not considered in our calculations.

In the simulations the master equations were solved with the procedure described by Johansson *et al*.^21^. As the first step, the density matrix *ρ* was re-casted to vector form as $\overrightarrow{\rho }$ ($\overrightarrow{\rho }=[{\rho }_{11},{\rho }_{21}\cdots ,{\rho }_{N1},{\rho }_{12}\cdots {\rho }_{NN}]$), and the equation of motion of the density matrix can then be re-written as

8 $$i\frac{d\overrightarrow{\rho }}{dt}=\overleftrightarrow{L}\overrightarrow{\rho }$$

all of the elements of the tensor $\overleftrightarrow{L}$ can be deduced from Eq. 2. Due to the time-dependent terms in the interaction Hamiltonian, the stationary state density matrix (${\overrightarrow{\rho }}_{S}$) will also be oscillating in time with the laser frequency as^21^

9 $${\overrightarrow{\rho }}_{S}={e}^{-i\overleftrightarrow{{\rm{\Omega }}}t}{\overrightarrow{\rho }}_{0}$$

where the time-dependent part of the density matrix is fully described by ${e}^{-i\overleftrightarrow{{\rm{\Omega }}}t}$ which leaves ${\overrightarrow{\rho }}_{0}$ time-independent. Here $\overleftrightarrow{{\rm{\Omega }}}$ is a diagonal tensor and only the diagonal elements referring to interband coherences are nonzero (equal to +*ω*_*in*_ and −*ω*_*in*_ for “excited-ground” and “ground-excited” coherences, respectively). By inserting Eqs 9 into 8 and multiplying both sides with ${e}^{i\overleftrightarrow{{\rm{\Omega }}}t}$, we get

10 $$[{e}^{-i\overleftrightarrow{{\rm{\Omega }}}t}\overleftrightarrow{L}{e}^{i\overleftrightarrow{{\rm{\Omega }}}t}-\overleftrightarrow{{\rm{\Omega }}}]{\overrightarrow{\rho }}_{0}=0$$

Here ${e}^{-i\overleftrightarrow{{\rm{\Omega }}}t}\overleftrightarrow{L}{e}^{i\overleftrightarrow{{\rm{\Omega }}}t}$ is also time independent. In our simulations, Eq. 10 was solved numerically by computing the corresponding null space of the matrix ${e}^{-i\overleftrightarrow{{\rm{\Omega }}}t}\overleftrightarrow{L}{e}^{i\overleftrightarrow{{\rm{\Omega }}}t}-\overleftrightarrow{{\rm{\Omega }}}$ through complex singular-value decomposition (SVD).
